# Analysis of hybrid solutions with Li-ion battery system for DP-2 vessels

**DOI:** 10.1016/j.heliyon.2024.e37068

**Published:** 2024-08-30

**Authors:** Sankarshan Durgaprasad, Zoran Malbašić, Marjan Popov, Aleksandra Lekić

**Affiliations:** a Delft University of Technology, Faculty of EEMCS, Delft, the Netherlands; b Alewijnse, Krimpen aan den IJssel, the Netherlands

**Keywords:** Li-ion, Battery energy storage system (BESS), Diesel engine, MILP, Optimal sizing, Fuel saving

## Abstract

An economical approach for incorporating Battery Energy Storage Systems (BESS) onto DP-2 vessels is presented in this research. The paper deals with developing a Battery Optimization for Optimal Sizing and Throughput Energy Regulation (BOOSTER) framework for putting research findings into practice by optimizing battery size, technology choice and power generation scheduling while considering battery degradation. Twelve battery sizes are analyzed based on three key performance metrics: return on investment, payback period, and years of profitability. A Mixed Integer Linear Programming (MILP) is developed to operate the energy and power management system of the vessel in a fuel and economically efficient manner. The study considers two load profiles of a DP-2 vessel operating near Taiwan and the North Sea. Our findings emphasize the significance of taking battery ownership costs in the form of energy throughput cost and fuel price into account, resulting in a longer battery lifetime and higher return on investment. The research also proposes a BESS operation matrix that provides vessel operators with valuable information on BESS usage for economic benefits. This matrix translates analytics and decision-making into tangible actions that can be implemented in real-time operations. Based on the findings, energy systems may be optimized for a sustainable future, which benefits vessel operators and industry stakeholders.

## Highlights

• BOOSTER framework optimizes battery size, technology, and power generation for DP-2 vessels.
• MILP model ensures fuel-efficient and cost-effective hybrid system operations.
• Twelve battery sizes analyzed for ROI, payback period, and profitability metrics.
• BESS operation matrix guides vessel operators for optimal economic benefits.

## Graphical abstract

**Figure:**
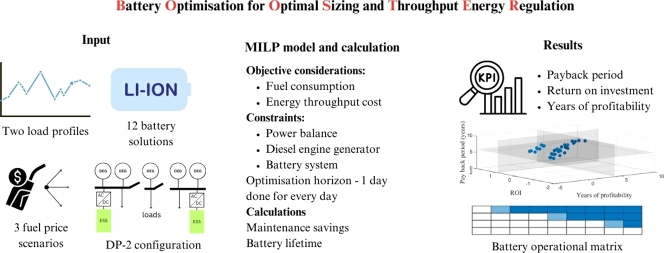

## 1 Introduction

As of 2018, the maritime industry is responsible for 1056 million tonnes of $CO_{2}$ in greenhouse gas emissions [1]. Compared to the 962 million tons of $CO_{2}$ generated in 2012, this is a 9.3% increase. Shipping emissions as a percentage of all anthropogenic emissions have grown from 2.76% in 2012 to 2.89% in 2018. The aim of the worldwide public to minimize greenhouse gas emissions has a significant impact on the design and operation of transportation infrastructure today. In 2018, the Marine Environment Protection Committee (mEPC72) of the International Maritime Organization approved the first-ever plan to reduce greenhouse gas emissions from global shipping. This IMO strategy reports a broad vision for decarbonization, greenhouse gas reduction targets through 2050, a list of short-, mid-, and long-term actions to accomplish these targets, obstacles to attaining the targets and supportive actions to overcome them, and criteria for future assessment. Abovementioned activities are summarized in [2].

Since the overwhelming success of the first fully electric ferry “The Ampere” in 2015, 70 other such ferries have shown profitability in Norway [3]. Experience shows that 127 out of 180 ferries are deemed to be profitable with either battery or hybrid operation [4]. The successful outcomes in Norway's ferry industry show that electric and hybrid propulsion technologies for maritime transportation are technically feasible and commercially viable. As a result, attempts are being undertaken to investigate how other types of vessels besides ferries may be electrified.

The primary objectives of this paper are to provide a battery system that is appropriately optimized, to ensure that the energy system functions effectively, and to provide the strongest possible business case. The study focuses on a DP-2 vessel that operates in the North Sea and Taiwan. The paper investigates the prospect of retrofitting the vessel with a battery system to transform it into a hybrid system. Retrofitting of vessels with BESS is usually performed by electrical system integrators. Therefore, it is necessary to analyze different solutions. Optimal sizing of the battery energy storage system is done by considering 12 different battery solutions from 2 European battery suppliers. These solutions include different battery technologies such as High Power or High Energy Li-ion batteries or a combination of both.

For vessel operators, integrating BESS has several operational benefits. The capacity to operate diesel engines at higher or more efficient points to maximize their performance is a significant advantage, especially for most vessels. Battery systems can also act as a “virtual generator” in the case of DP-2 vessels during DP mode, removing the need to operate numerous generators at low or inefficient operating levels. In addition to saving on fuel, this approach lowers the time of diesel engines and accompanying maintenance costs.

Hybridization of vessels does not terminate at integrating an optimally sized battery system. The existing power management system (PMS) and energy management system (EMS) must also be upgraded to function effectively. A BOOSTER (Battery Optimization for Optimal Sizing and Throughput Energy Regulation) methodology is proposed in this paper. The BOOSTER incorporates the operation of an optimized management system functioning based on the fuel price and the energy throughput cost (ETC) of the battery system.

The authors of this work focus on analyzing potential hybrid solutions for DP-2 vessels using Li-ion batteries. The contribution of this work is achieved through the combination of the following,

1. A methodology called BOOSTER is proposed to analyze different battery types and sizes for a DP-2 vessel. The analysis examines two key battery functions: facilitating optimal operation and serving as a reserve in DP operations. This is performed using a MILP model aimed at minimizing fuel consumption. Battery lifetime is calculated considering usage and calendar aging. The diesel engine maintenance savings are evaluated by analyzing the minimum time before overhaul (MTBO) of the diesel engine.
2. The best battery system is then chosen based on 3 key performance parameters and operated in an economically efficient manner, with the MILP model additionally considering the ETC of the battery. This ensures the battery is used only when it is economically advantageous, not just to save fuel. Three different fuel price scenarios are evaluated.
3. To support this economical operation, a BESS operational matrix is provided as guidance for vessel operators and the energy-power management systems.

These contributions provide a holistic approach to evaluating the feasibility of a battery system for a DP vessel and translate economic operation into actionable steps through the proposed BESS operational matrix. The contributions mentioned highlight the following novelties of this work,

1. Unlike current state-of-the-art methods that size components and determine their number based on optimization techniques, the proposed methodology relies on a design space derived from engineering experience and realism. This approach ensures more practical and feasible solutions tailored to real-world applications.
2. The study uniquely incorporates the MTBO in its analysis, which is not commonly considered in other DP-2 vessel studies. Additionally, it evaluates maintenance based on energy throughput and how the diesel engine is used, rather than just running hours. This comprehensive evaluation includes ETC in the objective function, simplifying the optimization process by avoiding multi-objective optimization and expressing objectives in the same cost units.
3. The proposed methodology and model offer a holistic approach by considering both technical and economic factors in a combined framework. This simplicity and combination present a novel contribution towards making the optimization process more straightforward and applicable to practical scenarios.

The parts of the paper are structured as follows. An overview of the existing literature covering optimization methods for BESS implementation in vessels, battery degradation and diesel engine generator (DG) maintenance is discussed in section 2. A brief overview of the existing DP-2 vessel is provided in Section 3. Section 4 provides the methodology, formulates the MILP problem, and details the simulated cases. The optimization problem results for both Taiwan and the North Sea are showcased in Section 5, through a front of optimal solutions and a viable business case with the BOOSTER is presented. Finally, Section 6 provides concluding remarks.

## 2 Implementation and optimization of BESS

### 2.1 Optimum operation of vessel management system and battery sizing

Optimization of EMS and storage system sizing for vessels have been thoroughly explored in the literature. For example, in [5] the authors have used linear and quadratic programming to optimize the sizing of the carbon capture and energy storage system and the vessel EMS. The importance of combining carbon capture and BESS was highlighted to reduce greenhouse gas emissions by 10% to 60%, with a corresponding increase in operational costs of 6.8%. In [6], nonlinear programming was used to optimize the shipboard BESS, where the authors split the operational profile into various modes and considered reactive power flow. The optimal size of the DG's for different operating states was determined using the Branch and Bound technique in [7]. This approach can also be extended to determine the ideal size of a BESS. Comprehensive optimization of the vessel EMS and BESS sizing was conducted in [8] using the OBLIVION framework, which considers safety constraints, vessel operating modes, sensitivity analysis, and battery degradation. The authors use energy throughput to predict the battery's lifetime and to limit the energy that flows through the battery system over its lifespan. Mixed Integer Nonlinear Programming (MINLP) was used in [9] and [10] to examine effective ship system planning, operation, and battery sizing. Dynamic programming was used in [11] and [12] for fuel savings through the generator, speed, and distance optimization. Finally, [11] achieved optimal power while considering a BESS by varying ship speed, and [12] presented a multi-objective mathematical programming model for optimized energy dispatch considering emissions, energy balance, and technical constraints.

Meta-Heuristic optimization utilizes several optimization methods, including Particle Swarm Optimization (PSO), Genetic Algorithms (GA), NSGA II, and Improved Sine and Cosine Algorithms (ISCA). PSO is used in [13] to optimize the scheduling of diesel generators in a DC-based off-shore support vessel, resulting in a reduced fuel consumption of 307 tons annually when compared to an AC architecture. The authors of [14] use a modified fuzzy-based PSO to model a ferry power management system that focuses on reducing emissions and operating costs. In [15], GA is employed to solve a mixed integer nonlinear problem that minimizes the power generation cost by optimizing the vessel's installed capacity and the pump loads. The authors also consider the generator's operational efficiency regarding power factor and loading percentage, which was not done in previous studies. These optimization techniques utilize various power management tactics to fulfill restrictions and reach optimization goals, resulting in improved convergence and optimal solutions. The authors of [16] use the ISCA algorithm that yields more optimal results than other evolutionary algorithms.

A technique described in [17] uses double-layer optimization to improve decision-making for investment and sizing. The inner loop uses MILP, and the outer loop uses NSGA-II. It was applied to retrofit a crew transfer vessel, minimizing investment, operation, and fuel costs. The authors considered different battery and fuel costs, presenting their findings through a Pareto front. A two-layer optimization approach has been proposed in a similar study [18]. The outer layer, utilizing NSGA-II, estimates the capital expenditure costs incurred. In contrast, the inner layer targets optimizing the EMS to minimize operational expenditure. According to the results, implementing a BESS alone can reduce emissions by 10%, but a fuel cell and shore connection are necessary to achieve further reduction. Table 1 summarizes other optimization studies considering EMS-PMS optimization and storage sizing.

**Table 1:** Other vessel optimization studies.

Reference	Method	ES Sizing
[16]	ISCA	FC, BS
[19]	Fmincon	SC
[20]	MO-PSO	SC, BS, FW, MES
[21]	MO-DEA	BS
[16]	ISCA	FC, BS
[22]	NLP, MILP	BS
[23]	No Info	BS
[24]	IO	BS
[25]	Rule-Based	BS
[26]	MINLP	BS

FC - Fuel Cell, BS - Battery System (Chemical), SC- Super Capacitor, FW-Flywheel, Magnetic Energy Storage, MO-Multi Objective, DEA - Differential Evolution Algorithm, Interval Optimization, ISCA - Improved Sine and Cosine Algorithms

The model used in this paper employs MILP because of the availability of mature solvers, its predictable performance, and the assurance of achieving a global optimum.

### 2.2 Battery degradation and optimization

Six essential aspects must be considered when retrofitting a battery in a vessel. These include the price, safety, and physical characteristics such as size and weight, as well as the battery's operating performance, encompassing capacity, power, and lifespan. The importance of each factor varies depending on the application. In the maritime industry, the capacity and power rating of the battery affect the ship's range and speed, while the lifespan and cost determine the expenses associated with installation and operation. There are two primary battery types used in the maritime industry, nickel manganese cobalt (NMC), and lithium iron phosphate (LFP). A list of maritime battery suppliers has been attached to the supplementary material. NMC batteries offer higher specific energy but come at a higher cost, while LFP batteries have higher specific power, safety, and a longer lifespan [27], [28], [29]. Thus, it is crucial to determine the right technology and supplier, as it will dictate the constraints in the optimization model. In this paper, the reviewed literature is limited to LFP and NMC batteries.

Battery degradation can be categorized into cycle aging and calendar aging. Cycle aging of the battery system refers to the degradation and the subsequent loss of battery capacity due to repeated cycling of the batteries. Several studies have been performed on modeling battery systems for NMC in [30], [31] and LFP batteries in [32], [33], [34]. Several studies have been conducted on the impact of C-rate and DoD on battery lifespan. In particular, the study in [30] looked at 21 batteries and five different C-rates, while [31] examined 12 batteries and 4 C-rates. In both studies, it was found that C-rate severely affects NMC batteries. On the other hand, [33] and [34] analyzed three batteries with 2 C-rates and 200 batteries with 4 C-rates respectively, and concluded that for LFP batteries, the critical degradation factor is DoD and not C-rate below 4C. The authors of [32] performed a similar analysis on one battery over 4500 cycles at three different C-rates and came to similar conclusions. Considering these variations when modeling the optimization problem or determining the appropriate battery size is essential.

Calendar aging of the battery refers to the degradation that takes place irrespective of the battery system cycling. Research on calendar aging has been a major area of focus within the electric vehicle field. This is because their batteries remain inactive for more than 90% of the time, as indicated in [35]. The authors of this study have thoroughly analyzed the impact of cycling and calendar aging on 258 cells for two different types of NMC batteries. The authors of [36] perform tests for calendar aging with 3 different types of cells, i.e., nickel cobalt aluminum (NCA), NMC, and LFP cells. Storage temperature affected calendar aging in 16 state of charge (SOC) levels, but the SOC did not consistently reduce capacity. Plateau regions were found at 20-30% SOC. NMC and NCA batteries degraded significantly at 60% SOC, while LFP batteries did so at 70%. In the maritime industry, DP mode is commonly used, charging batteries to high SOC to act as backup generators during system failure. There are several methods of incorporating battery degradation into the mathematical optimization model as shown in Table 2.

**Table 2:** Review of existing battery degradation modeling in optimization.

Reference	Method	Technique
[37]	MO-PSO	Semi-empirical & arrhenius
[38]	GA	Loss due to cycles
[39]	LP	Cost per kWh, DoD reduction
[40]	LP	Modified Shepherd Equation
[41]	MIP	Limit cycling
[42]	MINLP	DoD and Floatlife
[43]	LR	Incomplete and complete DoD
[44]	RHC	Discharge per cycle
[45]	EA	Cycles to failure
[46]	MILP	RCA

MIP - Mixed Integer Programming, LR - Linear Regression, RHC - Receding Horizon Control, RCA - Rain-flow counting algorithm

Various techniques have been suggested for precise cell/module level modeling in [37], [40], and [38]. However, obtaining the necessary parameters for these models from BESS suppliers can be difficult, making it challenging to model for retrofitting during systems integration. In [39], a linear programming method for off-grid power systems is used, considering the cost per kWh in the optimization model and the number of cycles to failure. The authors of [41] limit the total number of cycles the battery can perform based on a fixed cycle count over its lifetime while achieving the same amount of renewable energy penetration. In [42], the authors provide a more economic solution for a period of 10 to 15 yeas by considering BESS degradation cost and associated investment costs. The model incorporates a linear approximation of the battery's deterioration per cycle and optimizes the battery system for each time step using the Receding Horizon Control scheme. Two sources, [43] and [46], introduce the “rain-flow” cycle counting algorithm to distinguish between complete and incomplete cycles. [43] and [46] employ linear regression and piece-wise modeling approaches, respectively, to prevent non-linearity and obtain more optimal solutions in BESS sizing models.

### 2.3 Economic implications of diesel engine operation

**Figure 1:**
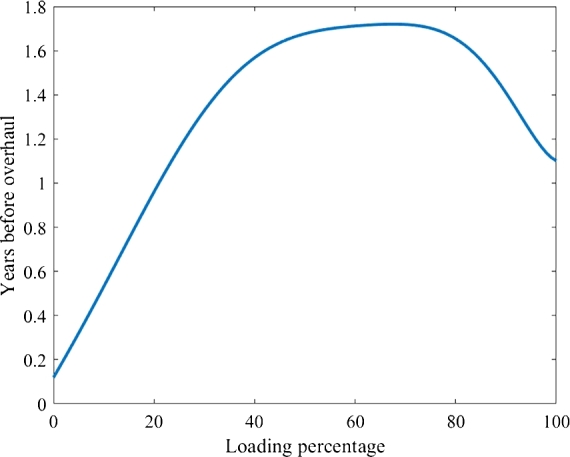
MTBO of diesel engine set.

To the best of the author's knowledge, there has been limited research on the economic impact of enhancing the loading percentage of DG sets. However, by validating vessel operators fleet maintenance records and using the authors approach in [47], additional maintenance savings can be realized through an extended time before overhauling diesel engines. This is graphically represented in Fig. 1, which depicts the minimum time in years before the DG needs an overhaul based on the loading percentage. It can be observed that the overhaul time is significantly less if the DG is loaded less than 40 percent and more than 85 percent. This function can mathematically represent an eighth-order polynomial function with the coefficients shown in Table 3. The dimensions of the coefficient $b_{i}$ are represented by $\frac{Hours}{{\left(kWh\right)}^{i}}$.

**Table 3:** MTBO parameters from [47].

Coefficient	Value
*b*_0_	1040.898
*b*_1_	3.429 × 10^4^
*b*_2_	1.66 × 10^4^
*b*_3_	4.971 × 10^4^
*b*_4_	−3.226 × 10^4^
*b*_5_	−5.504 × 10^5^
*b*_6_	2.803 × 10^6^
*b*_7_	−3.174 × 10^6^
*b*_8_	1.152 × 10^6^

The function is the summation of each coefficient $b_{i}$ multiplied with the loading percentage $\theta_{i}^{i}$. The authors of [47] claim that the costs for overhauling can be as high as 50% of the diesel engine cost, a similar ballpark number was provided by the vessel owner.

## 3 The vessel

**Figure 2:**
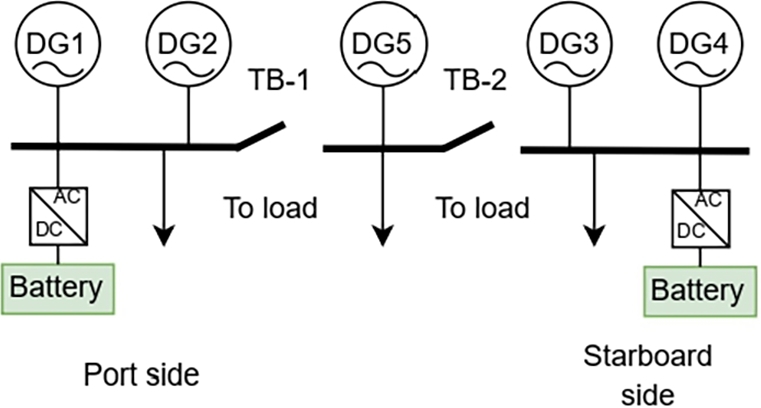
DP-2 vessel.

The single-line diagram of the analyzed vessel is shown in Fig. 2. The vessel comprises 5 DG's that are connected to a 690 V AC bus. The AC Bus is further separated into 3 segments using 2 bus tie-breakers (TB1 and TB2). DG1 and DG2 comprise the DG's present on the port-side (PS) of the vessel that is isolated from other DG's when TB1 is open. DG3 and DG4 are on the starboard (SB) side of the vessel and are isolated from the system when TB2 is open. DG5 is present in the middle busbar that is isolated from the system when TB1 and TB2 are open. The distribution network is connected to the main AC bus bar.

Based on the status of the Bus Tie breakers, the vessel operates in different modes. I.e. in DP mode, both the tie-breakers are open, isolating the PS, SB, and the middle section with DG5. When TB1 is open, and TB2 is closed, the vessel is considered to be in Non-Critical DP (NCDP01) mode. The notation NCDP10 applies when TB2 is open, and TB1 is closed. When TB1 and TB2 are closed, the vessel is said the be in Auto-Mode. It is expected to be redundant concerning the number of generators operating during DP mode in case of failure. Table 4 displays the power ratings and SFOC of the DGs. The SFOC coefficients are represented by *α* and *β*.

**Table 4:** Review of existing battery degradation modeling in optimization.

DG Number	Power (kW)	*α*, in $\frac{\text{L}}{\text{kWh}}$	*β*, in $\frac{\text{L}}{\text{h}}$
1,4	1912	0.1918	33.778
2,3	2560	0.1869	54.9209
5	1530	0.2351	20.024

**Figure 3:**
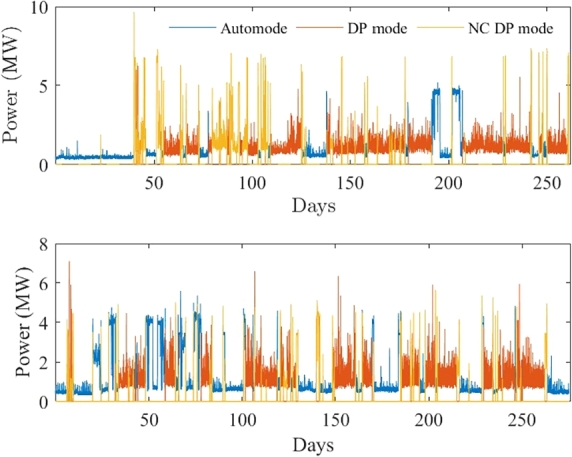
North Sea and Taiwan load profile.

This particular vessel is operational in two separate bodies of water: Taiwan and The North Sea. The recorded data for these two operational profiles span 256 (five minutes sample time) and 286 days (one minute sample time), respectively, as depicted in Fig. 3.

## 4 Methodology, solution space and MILP formulation

### 4.1 Methodology

**Figure 4:**
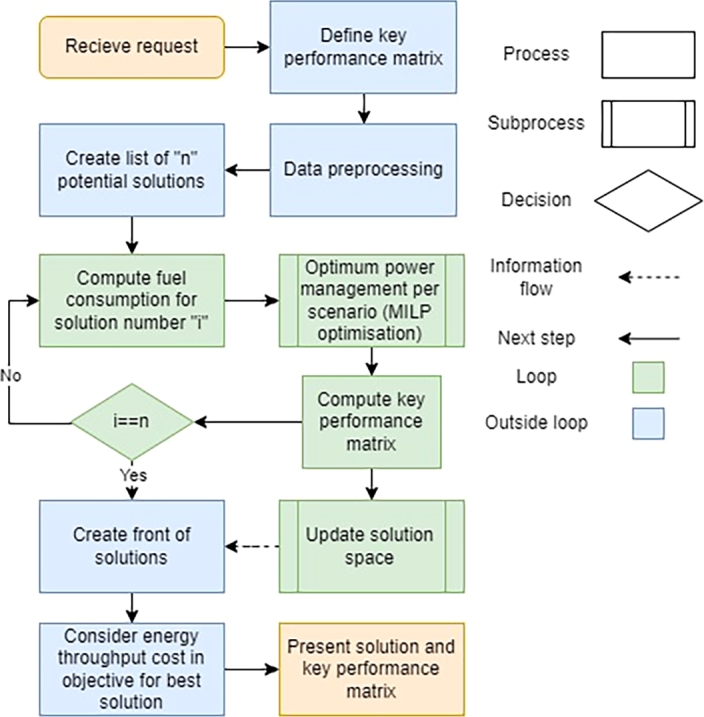
Methodology.

Fig. 4 illustrates the methodology used. The process initiates when a request for vessel hybridization is received. The customer's concerns are identified, and a key performance matrix (KPM) is defined, consisting of a list of key performance parameters. In this study, KPM includes return time of investment (ROI), payback period, and years of profitability, as discussed in later sections. The data is pre-processed in the next stage to ensure its usability. This is followed by developing a solution space of *n* (n=12) possible solutions. These solutions are then implemented in the model, and used for energy system optimization using MILP. The system is optimized for a time horizon of one day and repeated daily for the entire load profile. Operational expenses are evaluated by considering fuel and maintenance savings, and key performance parameters are subsequently assessed with respect to battery lifetime and capital costs. After obtaining the results, they are presented in a “front of solutions” and discussed with the customer. The KPM is then fine-tuned according to their specific requirements. The BOOSTER is implemented for the best solution where the ETC is considered in the objective function and its impact is analyzed accordingly.

The advantage of the proposed methodology is as follows,

1. The proposed methodology relies on an initial solution space of potential battery solutions for hybridization. This approach avoids the use of non-linearity in component sizing and determining the number of components.
2. The proposed solution space for DP-2 vessels is based on the potential functions of the battery. Similar solution spaces can be created for other hybrid vessels, whether the goal is to replace a DG or reduce the size of a DG during the initial design phase. The rest of the methodology can be followed in the same manner as for DP-2 vessels.
3. The methodology accounts not only for the fuel savings from the presence of batteries on board but also for savings from operating DGs at efficient points and turning off DGs during DP operations.
4. The methodology accounts for battery system aging outside the optimization framework, reducing the computational burden caused by the non-linearity of battery degradation.
5. Each solution is presented and its KPIs are evaluated, providing a robust design space for decision-making.
6. The best solution is economically optimized by considering energy throughput costs, enabling the creation of actionable steps through the BESS operational matrix. This provides vessel operators and energy management systems with clear guidelines for efficient and cost-effective operation.

The proposed methodology has the following limitations,

1. The methodology is highly dependent on the initial solution space created.
2. Calculating battery degradation outside the optimization framework prevents degradation-aware operation for all solutions. This consideration is only partially addressed for the best solution, where ETC are included in the objective function.
3. Calendar aging is fixed at 3% for every year that the battery is not used. Though it can vary depending on usage patterns. This variability is not considered.
4. The cost of battery usage is evaluated based on its energy throughput, which does not account for unequal charge and discharge cycles.
5. This methodology is not relevant to vessels with fuel cells, as it calculates maintenance savings based on the MTBO curves of a diesel engine. Additionally, battery sizing for fully electric vessels is not performed in the same manner. The current method and model are designed for hybrid systems where battery charging can take place onboard the vessel.

### 4.2 KPM and solution space

The KPMs used in this paper to evaluate the performance of each solution are ROI, payback period, and the years of profitability (YOP). These are explained by equations (1)-(3).

(1) $$ROI=\frac{Profit-Initial\;investment}{Initial\;investment}$$

(2) $$Payback\;period\;(years)=\frac{Initial\;investment}{Profitperyear}$$

(3) YOP(years)=Batterylifetime−Paybackperiod

The values of the following performance indicators are set to

PaybackTime≤6years,YearsofProfitabilityTime≥4years,ROI≥0.9.

The battery lifetime and profit are provided by equations (4) and (5). The profit is divided into two parts, fuel savings, and maintenance savings.

(4) $$\frac{Total\;number\;of\;cycles}{Cycles\;per\;year+calendar\;aging(cycles\;equivalent)}$$

(5) Profit(Euro)=FuelSavings+MaintenanceSaving

Equation (6) shows how maintenance savings (Euros) are calculated. Here, $Cdg_{n}$ denotes the cost of the DG, *T* represents the total number of periods, $\Theta_{itn}^{i}$ indicates the current loading percentage of generator *n* at time *t* in Euros, and *i* refers to the exponential power. On the other hand, $\theta_{itn}^{i}$ represents the optimized loading percentage of generator *n* at time *t*, where *i* is the exponential power. The coefficient $b_{i}$ can be obtained from Table 3. At any given time, the loading percentage of the DG can be calculated by dividing the actual power by its rated power. The fuel savings calculation method is discussed in subsection 4.3.

(6) $$MTBO\;Savings=\sum_{n=1}^{5}\left(0.5\times Cdg_{n}\left(\frac{\sum t=1^{T}(\sum_{i=1}^{7}(\Theta_{itn}^{i}-\theta_{itn}^{i})\times b_{i})}{T}\right)\right)$$

The equations in this subsection are not included in the optimization process. Instead, they are solved using the results from the optimized PMS-EMS system outlined in subsection 4.3. Table 5 presents the solution space for this work. Solutions 1-9 are designed to replace DG1 and DG4 during DP operations, acting as a reserve, while solutions 10-12 are intended for use during Non-DP operations. The criterion for a solution to function as a reserve is that it must be able to provide 80% of the power of DG1/DG4 in order to turn them off during DP operation. Table 5 also shows the investment cost of batteries, the number of cycles, and the energy throughput costs associated with each kWh the battery discharges.

**Table 5:** Solution space.

No.	Capacity (Netto)	DoD (%)	Cost (Million)	Cycles	TC $\left(\frac{Cost}{kWh}\right)$
1	1530 x2	70	2.18	10000	0.05
2	1530 x2	75	2.04	7500	0.0667
3	1530 x2	80	1.91	5000	0.1
4	510 x2	70	1.16	12133	0.0659
5	510 x2	75	1.08	9166	0.0873
6	510 x2	80	1.02	6200	0.129
7	1000+175 x2	70	1.56	10000	0.0548
8	1000+175 x2	75	1.6	7500	0.0803
9	1000+175 x2	80	1.52	5000	0.121
10	1000 x1	70	0.7	10000	0.05
11	1000 x1	75	0.6	7500	0.0667
12	1000 x1	80	0.62	5000	0.1

Cost represented in Euros, cycles represent the number of cycles they can endure till 80% of capacity remaining, TC - Throughput cost

### 4.3 MILP formulation

As stated earlier this research uses MILP to formulate the optimization problem. The linearity of both the objective function and constraints allows for the use of this optimization technique. Furthermore, MILP problems have a more structured form, which is advantageous for modeling, analysis, and interpretation. The decision variables and constants used in the optimization are depicted in Table 6, Table 7.

**Table 6:** Decision variables used.

Notation	Description	Variable
*u*_*it*_	*DG*_*i*_ Status	Integer
*PG*_*it*_	*DG*_*i*_ Power N	Continuous
*Ebat*_*t*_	Energy stored in battery	Continuous
*Ton*_*it*_	Minimum on time of *DG*_*i*_	Integer
*Ebatcharging*_*t*_	Charging Energy of Battery	Continuous
*Uon*_*it*_	*DG*_*i*_ Turn-On	Integer
*δ*_*it*_	Parallel Loading of DG	Integer
*M*_*t*_	Battery Charging	Integer

UC - Unit commitment

**Table 7:** Constants used.

Notation	Description
*C*_*i*_	*DG*_*i*_ Start up cost
*PG*_*min*_	DG minimum power
*PG*_*max*_	DG max power
*C*_*rate*_	Maximum C-rate
*PG*_*irated*_	DG rated power (Table 4)
*Ri*	Ramp rate of *DG*_*i*_
*TC*	Throughput cost of BS
*M*	Big M integer
*X*	Big M integer
*Max*_*NC*_	Maximum number of cycles
*η*	One way efficiency

#### 4.3.1 Objective function

The objective function's goal is to minimize the operational costs (OC), which can be expressed as:

(7) OC=FuelConsumption×FuelPrice+ETC.

The fuel consumption can be split into fuel consumed due to power generation ($F_{PG}$) and starting up the DG ($F_{SG}$). The fuel consumed due to power generation is shown by equation (8):

(8) $$F_{pg}=Price\;of\;Fuel\times \sum_{t=1}^{T}(\sum_{i=1}^{DG_{n}}(\alpha_{i}\times Pg_{it}+\beta_{i}))\times \Delta t.$$

The $F_{SG}$ can be linearly modeled using the big M integer method as shown in equations (9)-(11).

(9) $$u_{it}-u_{i(t-1)}\geq 1+0.001-M(1-Uon_{it}),$$

(10) $$u_{it}-u_{i(t-1)}\leq 1+M(Uon_{it}),$$

(11) $$F_{SG}=C_{i}\times \sum_{i}^{T}\sum_{i=1}^{DG_{n}}(Uon_{it}),$$

where $C_{it}$ is the startup and shutdown cost of $DG_{i}$. The value of $Uon_{it}$ holds the value of 1 every time $DG_{i}$ goes from on-state to off-state and 0 otherwise. The ETC can be modeled by summing the total amount of charging the battery undergoes during each cycle or partial cycle and multiplying it by the throughput costs from Table 5. This is shown in equation (12).

(12) $$ETC=TC\times \sum_{i}^{T}Ebatcharging_{t}.$$

#### 4.3.2 Constraints

The generators have upper limit (80% of rated power) and lower limit constraints (40% of rated power). These limits are based on the SFOC and diesel engine maintenance curves. The constraints are modeled as per (13), (14). The values of $PG_{min}$ and $PG_{max}$ are 0.4 and 0.8, respectively.

(13) $$PG_{it}\geq PG_{min}\times PG_{irated}$$

(14) $$PG_{it}\leq PG_{max}\times PG_{irated}$$

The DG's must also be associated with unit commitment ($U_{it}$) (ON-OFF state). These constraints are modeled through equations (15), (16):

(15) $$PG_{it}\geq U_{it}\times PG_{irated}\times PG_{min}$$

(16) $$PG_{it}\leq U_{it}\times PG_{irated}\times PG_{max}$$

The DG set is also constrained with ramp-up and ramp-down limits. The ramp limits are considered 20% of the maximum allowable power. Turning ON and OFF, the generators have no ramping limits. This is incorporated by adding a unit commitment term. Ramping up and ramping down limits are presented by equations (17), and (18), respectively. In equation (17), the variable $ui_{(}t-1)$ is 0 if the DG is turned on at time t, it is similarly done in equation (18). These are the conditions stated above.

(17) $$PG_{it}-PG_{i(t-1)}\leq ((0.3\times (1-u_{i(t-1)}))+Ri_{rate})\times PG_{irated}$$

(18) $$PG_{i(t-1)}-PG_{it}\leq ((0.3\times (1-u_{i(t)}))+Ri_{rate})\times PG_{irated}$$

When two or more DGs are ON, they are loaded parallelly, i.e., the load is shared between the DGs proportionally to their rated power. For example, parallel loading of DG1 - DG3 is modeled by equations (19)-(22).

(19) $$u_{2t}+u_{3t}\geq 1+0.001-M\times (1-\delta_{1t}),$$

(20) $$u_{2t}+u_{3t}\leq 1+M\times \delta_{1t},$$

(21) $$\frac{PG_{3t}}{PG_{3rated}}-M\times (1-\delta_{1t})\leq \frac{PG_{2t}}{PG_{2rated}}$$

(22) $$\frac{PG_{2t}}{PG_{2rated}}\leq \frac{PG_{3t}}{PG_{3rated}}+M\times (1-\delta_{1t})$$

When both DG1 and DG3 are switched on, the $\delta_{1t}$ value is set to 1. In this case, the variable M is a large integer with a value of 8000. To minimize the number of constraints and variables, parallel loading of only a few selected DGs is performed due to the similarities between $DG_{1,4}$ and $DG_{2,3}$, and because the power demand in the load profiles does not require the full installed capacity on board.

The minimum ON-time ensures that the generators are on for a minimum specific duration. This is described by the following equation:

(23) $$\sum_{t=1}^{Min\;Time}(U_{it})=Min\;Time\times Ton_{i}\;\;\;\;\;\vee T.$$

Here $Ton_{i}$ is a Boolean decision variable ensuring that the sum of the unit commitment variable $U_{it}$ is either ON for the minimum specified duration or OFF. The value of minimum ON-time is set to 20 minutes.

The net capacity of the battery system serves as the basis for its modeling. When it is in an Auto mode, the energy storage system can be represented by the combined net capacity of the BESS on both the PS and SB side, denoted as $E_{max}$. The stored energy ($Ebat_{t}$) that can be utilized at any given time cannot exceed the net capacity of the combined BESS, and cannot go below zero. These parameters are mathematically modeled by the following equations:

(24) $$Ebat_{t}\geq 0,$$

(25) $$Ebat_{t}\leq E_{max}.$$

The minimum C-rate restriction ensures that the battery system charges and discharges within its technical capabilities. A single charging and discharging C-rate is considered. Charging and discharging are represented by the following equations (26) and (27), respectively.

(26) $$Ebat_{t}-Ebat_{t-1}\leq C_{rate}\times E_{max}\times \Delta t,$$

(27) $$Ebat_{t-1}-Ebat_{t}\leq C_{rate}\times E_{max}\times \Delta t.$$

It is necessary to calculate the total charge energy to determine the number of complete or partial charge cycles the battery goes through. As previously done, this can be achieved using the big M integer method. The variable $M_{t}$ is an integer that equals 1 when the battery is charging and 0 otherwise. Equations (28)-(31) outline the formulation of the decision variable $Ebatcharging_{t}$, which only includes the charged values of the battery.

(28) $$Ebatcharging_{t}\geq 0,$$

(29) $$Ebatcharging_{t}\geq Ebat_{t}-Ebat_{t-1},$$

(30) $$Ebatcharging_{t}\leq 0+M\times M_{t},$$

(31) $$Ebatcharging_{t}\leq Ebat_{t}-Ebat_{t-1}+M\times (1-M_{t}).$$

According to ((28)), the battery charging variable can only have values greater than 0 and the maximum possible amount of charge (kWh) for a given period. This is represented by equation (32).

(32) $$Ebatcharging_{t}\leq C_{rate}\times E_{max}\Delta t$$

Based on this, the number of cycles for a given period T can be computed as

(33) $$Number\;of\;Cycles=\frac{\sum_{t=1}^{T}Ebatcharging_{t}}{Emax}$$

The battery degradation or cycle limitation can be limited per time segment using inequality constraints as shown in the equation:

(34) $$\sum_{t=1}^{T}Ebatcharging_{t}\leq Emax\times Max_{NC}.$$

The energy flow or load balance equation is modeled by considering the round trip efficiency of the system *η*. There are two main equations, i.e. Charging, and discharging. While charging the battery, it is already established that the value of $M_{t}$ = 1 and 0 otherwise. Therefore, the energy balance equations for charging (equations (35), (36)) and discharging (equations (37), (38)) can be modeled as:

(35) $$Ebat_{t}\geq Ebat_{t-1}+\eta \times \Delta t\left(\sum_{t=1}^{T}PG_{it}-Pdemand_{t}\right)-X\times (1-M_{t}),$$

(36) $$Ebat_{t}\leq Ebat_{t-1}+\eta \times \Delta t\left(\sum_{t=1}^{T}PG_{it}-Pdemand_{t}\right)+X\times (1-M_{t}),$$

(37) $$Ebat_{t}\geq Ebat_{t-1}-\frac{\Delta t\left(Pdemand_{t}-\sum_{t=1}^{T}PG_{it}\right)}{\eta }-X\times M_{t},$$

(38) $$Ebat_{t}\leq Ebat_{t-1}-\frac{\Delta t\left(Pdemand_{t}-\sum_{t=1}^{T}PG_{it}\right)}{\eta }+X\times M_{t}.$$

Here *X* is a big integer equal to $8\times 10^{3}$. A round trip efficiency of 96% is considered, hence the value of ηis0.98.

### 4.4 Modes of optimization

The optimization is performed for the three modes of operations, i.e., the AUTO mode, non-critical DP (NCDP) mode, and DP mode. The MILP formulation's constants values are listed for each mode in Table 8.

**Table 8:** Mode dependent constant values.

Notation	Auto	DP & NCDP
*C*_*i*_	Rated SC	Rated SC
*PG*_*min*_	0.4	0.2
*PG*_*max*_	0.8	0.8
*C*_*rate*_	Rated C-rate	0
*PG*_*irated*_	Table 4	Table 4
*Ri*	0.5 NS; NA Taiwan	0.5 NS,; NA Taiwan
*TC*	Table 5	NA
*M*	8000	8000
*X*	8 × 10^6^	8 × 10^6^
*Max*_*NC*_	Table 5	0
*η*	0.98	0.98
*Pdemand*_*t*_	PS+SB	PS, SB

NA - Not applicable where the value is 0 or the constraint is disabled, PS+SB indicates the power demand of PS + SB combined; it is separate for DP/NCDP

Optimization is done separately for each mode of operation to ensure optimal performance. After each AUTO mode, the battery charge $Ebat_{it}$ is set to its maximum to be used in DP and NCDP modes. In AUTO mode, the power demand combines SB and PS demand, while in DP and NCDP mode, SB and PS sides are treated separately. Results are combined for each mode to produce overall optimization. The value of Δt=1/12 (5 minutes) for Taiwan and Δt=1/60 (1 minute) for The North Sea.

In addition to the three different optimization modes, three different fuel price scenarios are also considered, as shown in Table 9. Therefore, we finally obtain results for 12 different solutions per Table 5 and for three different scenarios as shown in Table 9.

**Table 9:** Fuel price per scenario.

Scenario	Percentage of time		
Number	450 Euro/ton	650 Euro/ton	850 Euro/ton
1	33	50	17
2	50	33	17
3	50	50	0

## 5 Results

The combined optimized fuel savings per solution without considering the ETC costs are shown in Table 10. The optimized solutions also yield an increase in the MTBO of DG as shown in Table 11 and a subsequent decrease in the running time of DG as shown in Table 12. It is important to note that these results are obtained by applying the MILP optimization.

**Table 10:** Fuel savings per scenario.

Solution Number	Fuel Savings (tons)	Number of Cycles
Taiwan		
1-3	425.08	289.7
4-6	424.9	644.7
7-9	416.3	400
10-12	98.3	652.2

North Sea		
1-3	470.9	357.1
4-6	467.3	900.9
7-9	459.6	554.1
10-12	152.6	925.5

**Table 11:** Minimum time before overhaul (days).

Solution	MTBO (days)				
Number	DG1	DG2	DG3	DG4	DG5
Taiwan					
1-3	442.6	463.4	464.7	398.3	124.8
4-6	443.5	498.4	436.9	398.3	124.8
7-9	443.5	494.8	436.9	398.3	124.8
10-12	354.7	297.9	228.8	296.2	125.1
Current Scenario	326.9	298.8	250.4	326.2	130.4

North Sea					
1-3	486.7	603.3	619.6	414.7	151.3
4-6	505	603.1	619.2	414.7	151.3
7-9	495.2	603.0	619.2	414.7	151.3
10-12	452.1	440.1	369.7	378.4	151.1
Current Scenario	378.9	393.6	339.7	406.3	303.8

**Table 12:** DG running time.

Solution	Running time (days)				
Number	DG1	DG2	DG3	DG4	DG5
Taiwan					
1-3	174.3	56.3	58.2	169.3	134.2
4-6	175.3	72.5	48.4	169.3	134.2
7-9	175.3	70.4	48.4	169.3	134.2
10-12	185	122.2	116.9	147.3	134.4
Current Scenario	187.4	123.5	124.4	188.5	136.1

North Sea					
1-3	145.4	57.5	34.4	127.5	115.6
4-6	168.5	67.3	21.1	127.5	115.6
7-9	155.4	77.7	21.1	127.5	115.6
10-12	140.9	111.3	87.6	79.9	115.7
Current Scenario	145.3	91.6	100.2	107.2	183.4

Equation (6) is used to determine the maintenance savings. Additionally, the battery's expected lifespan is calculated by equation (4). The results of this analysis are presented in Table 13, which shows the annualized figures. Based on the fuel prices per scenario (Table 9), the payback period of each solution and the ROI is calculated by (3), (1) and presented in the supplementary material.

**Table 13:** Annualized result of fuel saving maintenance savings battery life time.

Annualized average result (Taiwan + North Sea)				
Solution number	Fuel savings (tons)	Maintenance savings (Euros)	BS life time (years)	Investment cost (Million Euros)
1	604.5	101863	13.6	2.68
2	604.5	101863	11.36	2.54
3	604.5	101863	8.54	2.41
4	601.5	100472	8.67	1.66
5	601.5	100472	6.99	1.58
6	601.5	100472	5.07	1.52
7	590	100706	10.64	2.06
8	590	100706	8.67	2.1
9	590	100706	6.33	2.02
10	167.5	16000	7.37	0.88
11	167.5	16000	5.85	0.82
12	167.5	16000	4.14	2.06

It is important to mention that the payback period, years of profitability, and ROI are calculated in the MILP optimization's outer loop. Based on these results, the solution with number seven offers the best performance based on the KPM parameters set in section 4.2. Therefore, the booster methodology, including ETC costs, is implemented by applying the seventh solution (BOOSTER 7) for the three scenarios. The KPM performance of BOOSTER 7 is presented in Table 14.

**Table 14:** Solution 7 BOOSTER performance.

Key performance index	Scenario number		
	1	2	3
Payback period	5.2	5.5	5.7
Years of profitability	8.3	9.6	9.7
ROI	1.6	1.78	1.5

**Figure 5:**
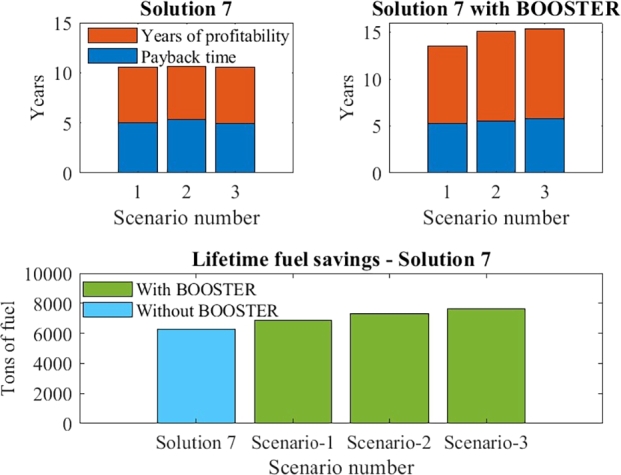
Payback period and years of profitability with and without BOOSTER.

Fig. 5 visually represents the comparison between Solution 7 with and without the BOOSTER. The BOOSTER optimization resulted in a significant increase of 21.88%, 81.63%, and 32.67% in ROI for Solution 7. This was made possible by the EMS-PMS's mindful operation, incorporating ETC and purchasing fuel price. Although the overall fuel savings per year were reduced in the BOOSTER method, the number of years of profitability increased, leading to higher lifetime fuel savings. This is due to disproportional fuel savings seen in DP mode. For Scenario 1, there was a 9.5% increase in lifetime fuel savings, and 16.4% and 21.3% increases for Scenarios 2 and 3, respectively. Another benefit of extending the battery lifetime is the annual savings on maintenance costs for more years.

**Figure 6:**
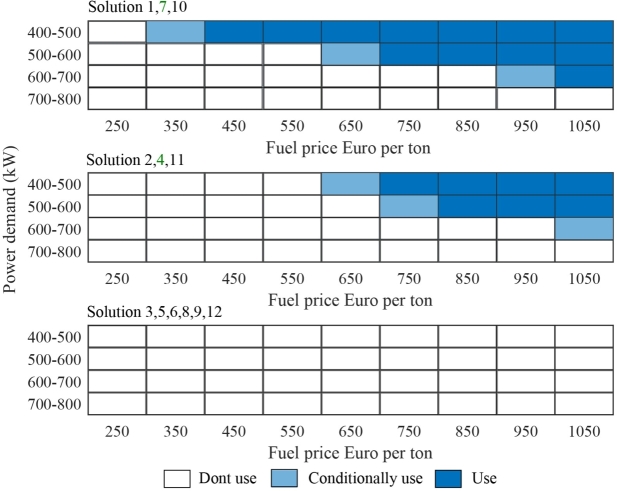
BESS operation matrix.

Fig. 6 shows the operational matrix of the BESS for the given power system network. The fuel cost and power demand are considered when deciding whether to use the battery system. Batteries with 70% DoD have a higher operational region as compared to others. It is recommended to choose solution 7 due to its low throughput cost to mitigate the risks linked with unstable fuel prices.

**Figure 7:**
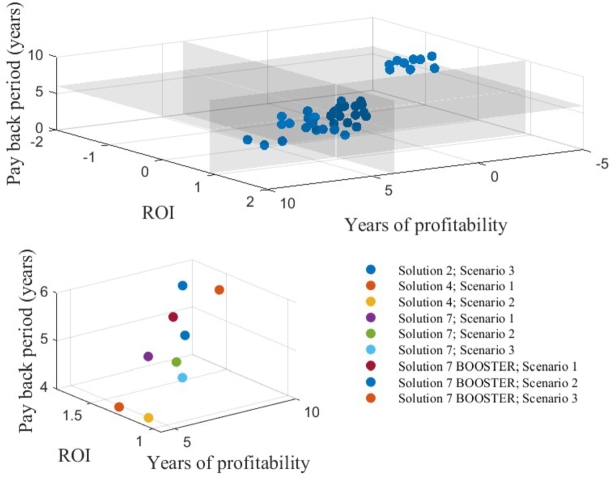
Front of all solutions for all scenarios.

Fig. 7 (top) provides a front/overview of all the solutions. However, not all these solutions align with the key performance indicators reported in section 4.2. Therefore, on applying the KPM boundaries, the solutions are presented in Fig. 7 (bottom), and these solutions are known as lucrative solutions. The front with lucrative solutions clearly shows the increase in the ROI when implementing the BOOSTER. In addition, high-power solution four is also feasible in the case of fuel price Scenario 1 and Scenario 2. Solution 2 is only feasible in the case of fuel price Scenario 3.

## 6 Conclusions

The research presented in this paper highlights the importance of a smart EMS-PMS system that incorporates the BOOSTER methodology. Rather than relying on a static average fuel price, the BOOSTER methodology considers fuel prices as a function of time, allowing the EMS-PMS to operate realistically in real-world vessel functioning. This includes knowledge of fuel prices, power requirements for various tasks, and the ETC of the battery or a decision to invest. There is also a disparity in the fuel savings per cycle observed between the results in the North Sea and Taiwan due to the higher power requirements in the North Sea. This further strengthens the need for a smarter management system.

A combination of HP and HE batteries is a cost-effective solution for vessel owners. HE batteries are cheaper per kWh than HP batteries, however, their large size to meet DP class requirements can be expensive. Therefore, combining HP + HE batteries is a better option as it requires a smaller battery size to meet class requirements. Additionally, the power electronic costs of the HP+HE system are the same as those for HP or HE systems, and lower-powered power electronic converters are cheaper than one large high-power converter. A considerable amount of fuel savings can also be observed due to overhaul maintenance savings of the DG. This is often overlooked while calculating or estimating an investment's feasibility.

This work has its limitations, and it is important to note that the current battery system experiences a static 3% calendar aging (year by year). However, the research conducted on how SOC, temperature, and cycling affect calendar aging is limited. Proper cycling of the battery can help reduce calendar aging, which is essential for the BOOSTER solution's longevity, particularly when it is supposed to be used for more than 10 years. To ensure that future developments of this model are successful, calendar aging must be considered concerning cycles, idle time, and SOC state. The costs of implementing the BESS consider the power electronic and battery system costs. Another crucial consideration in calculating investment costs is the expense of system integration. Due to the numerous factors that affect it, such as the number of hours required to upgrade the current PMS-EMS and space limitations on board, this has been deliberately excluded from the analysis. Additionally, the authors acknowledge their lack of knowledge regarding future interest rates, inflation rates, and fuel prices when this research has been conducted due to ongoing geopolitical and financial changes. As a result, these factors were excluded from calculating the payback period and ROI to maintain simplicity.

The paper proposes a methodology for fleet owners and system designers to make decisions and implement associated investments in BESS. Based on the obtained results, it is recommended to implement either Solution 4 or Solution 7, and strongly advocate for implementing the smart BOOSTER EMS-PMS system.

## Funding statement

This research did not receive any specific grant from funding agencies in the public, commercial, or not-for-profit sectors.

## CRediT authorship contribution statement

**Sankarshan Durgaprasad:** Writing – original draft, Visualization, Validation, Software, Resources, Project administration, Methodology, Investigation, Formal analysis, Data curation, Conceptualization. **Zoran Malbašić:** Supervision. **Marjan Popov:** Supervision. **Aleksandra Lekić:** Writing – review & editing, Supervision.

## Declaration of Competing Interest

The authors declare the following financial interests/personal relationships which may be considered as potential competing interests: Sankarshan Durgaprasad reports administrative support and writing assistance were provided by Alewijnse B.V. If there are other authors, they declare that they have no known competing financial interests or personal relationships that could have appeared to influence the work reported in this paper.

## Data Availability

The data used in this study has not been deposited into a publicly available repository, as it is confidential.

## Appendix A Supplementary material

Supplementary material Battery manufacturers and final solutions table. Supplementary material
